# Factors associated with the acceptance of mass drug administration for the elimination of lymphatic filariasis in Agusan del Sur, Philippines

**DOI:** 10.1186/1756-3305-1-14

**Published:** 2008-05-27

**Authors:** Maria Lourdes E Amarillo, Vicente Y Belizario, Jewel T Sadiang-abay, Stephanie Anne M Sison, Ariane Marie S Dayag

**Affiliations:** 1 Department of Clinical Epidemiology, College of Medicine, University of the Philippines Manila, 547 Pedro Gil St. Ermita, Manila 1000, Philippines; 2 Department of Parasitology, College of Public Health, University of the Philippines Manila, 547 Pedro Gil St. Ermita, Manila 1000, Philippines; 3 Filariasis Study Group, National Institutes of Health, University of the Philippines Manila, 547 Pedro Gil St. Ermita, Manila 1000, Philippines; 4 Filariasis Study Group, National Institutes of Health, Philippines

## Abstract

**Background:**

Mass drug administration (MDA) has been one of the strategies endorsed by the World Health Assembly for lymphatic filariasis (LF) elimination. Many factors, however, affect the acceptability of the MDA in the Philippines with acceptability defined as the ingestion of drugs -diethylcarbamazine and albendazole during MDA. These drugs were mainly distributed in fixed sites and mopping up activities were conducted through house-to-house visits to increase treatment coverage. The aim of conducting the study was to determine the MDA acceptance rate among a population endemic for LF, and the factors associated with MDA acceptance.

**Methods:**

In April 2005, a stratified cluster survey involving 437 respondents aged 18 years old and above in Agusan del Sur, Philippines was conducted. Key informant interviews and focused group discussions were performed among community leaders and health service providers. Descriptive statistics and coverage estimates were calculated with appropriate sampling weights applied to all analyses. Factors assessed for association with receipt of antifilarial drugs and MDA acceptance were respondents' socio-demographic characteristics, knowledge, attitudes, beliefs and perceptions on LF. Pearson chi-squared test was used to determine factors associated with MDA acceptance.

**Results:**

Results showed that 63.3% of the sampled population received the antifilarial drugs; of these, 94.5% ingested the drugs, yielding an acceptance rate of 60%. Half of the sampled population received the drugs from a fixed site, while only 13% was mopped up. A majority of the sampled population were aware of LF and MDA. Knowledge on LF prevention, cause, treatment and diagnosis and adverse events was low to moderate. Knowledge on LF and perceived benefits of antifilarial drugs were found to be associated with MDA acceptance (p = 0.08). Health workers remain the front liners in the MDA implementation. Local government units were aware of LF and MDA, but support was insufficient.

**Conclusion:**

The proportion of the sampled population that received and ingested the antifilarial drugs was much lower than the reported coverage. The target coverage rate of 85% may be achieved with sufficient groundwork for MDA, buy-in from the local government, greater efforts exerted to increase the people's knowledge on LF and MDA and their understanding of perceived benefits of the drugs. These would contribute to the successful elimination of LF in the province.

## Background

Lymphatic filariasis (LF) is a major cause of permanent disability and disfigurement worldwide. It affects about 120 million people and 1.2 billion people are at risk in over 83 countries worldwide [1]. In 1997, the World Health Assembly called for the elimination of LF as a public health problem. One of the main strategies to achieve elimination is a once a year mass drug administration (MDA) for four to five years to eligible population aged 2 years and older. The Communication for Behavioural Impact strategy developed by the World Health Organization is the currently recommended approach to implement MDA [2].

In the Philippines, the population at risk for filariasis is estimated to be 23.5 million people [3] in 40 endemic provinces with microfilaremia rates ranging from 0.05% to 29.2% [4]. The Department of Health aims to eliminate this disease through MDA using diethylcarbamazine and albendazole tablets given to the eligible population, mainly through fixed sites. Mopping up activity through house-to-house visits by volunteer health workers is usually done to cover the remaining target population who did not receive the drugs. Since the start of implementation in 2001, no study on MDA acceptability has yet been done. With the aim of scaling up coverage to all endemic areas, it is important to determine the acceptability of the MDA and identify factors that may be associated with it to help achieve the target coverage and ultimately eliminate the infection. Based on the Health Belief Model, demographic, predisposing, reinforcing, and enabling factors may affect the success of MDA for elimination of LF [5].

## Methods

The study was conducted in the province of Agusan del Sur, Philippines in April 2005, five months after the second round of the annual MDA. A cross-sectional survey, which applied a multi-stage stratified cluster sampling technique, was conducted. Four municipalities were randomly selected among the 14 municipalities in the province. From each municipality, three to six *barangays *(villages) were randomly selected with probability proportional to the estimated size [6] of the *barangay*. In each *barangay*, 25 to 29 individuals aged 18 years and above were randomly chosen from the MDA masterlist for 2004 through systematic sampling technique.

Face-to-face interviews were conducted by trained interviewers using a pre-tested questionnaire, which was translated to the local dialect and back-translated to English. Informed consent of each subject was obtained prior to the interview. In-depth key informant interviews among local health officers and community leaders were conducted. Focused group discussions were also done among field health personnel.

The study defined acceptance rate as the proportion of individuals in the sampled population who actually ingested the antifilarial drugs during the MDA in 2004. Compliance rate for the MDA round in the same year is defined as the number of people who reported that they ingested the drugs, divided by the total number of people who received the drugs multiplied by 100. Similarly, coverage rate is defined as the number of eligible population (aged 2 years and older) who reported that they ingested the antifilarial drugs, divided by the total number of eligible population multiplied by 100.

Potential factors that are thought may affect MDA acceptance include age, sex, socio-economic status, education, poor acceptability of ingesting many pills, knowledge and awareness, and attitudes, beliefs and perceptions about LF. Reinforcing factors such as satisfaction with health services, policy support from decision makers, and social support from family and community, as well as enabling factors such as availability and accessibility of diagnostic and treatment services, and self-efficacy or the confidence in one's ability to take action [7] may likewise affect MDA acceptance.

### Data Processing and Analysis

Data were encoded using CSPro 2.5 [8]. Data quality was ensured by performing double encoding and validation. STATA 8.0 [9] was used in data management and analysis. Sampling weights were calculated and applied in the analysis. Frequency and percent distributions were obtained for categorical variables, and the mean, standard deviation and median values of quantitative variables were computed where appropriate. A six-point Likert scale was used to quantify responses to attitude statements or items, with lower scores (1 to 3) representing agreement, and higher scores (4 to 6) representing disagreement. The degree of homogeneity (also called internal consistency or reliability) of the items in the attitude scale was determined by calculating the Cronbach alpha coefficient [10]. Total attitude scores were classified as either high or low.

Respondents' knowledge on cause, symptoms, diagnosis, treatment, and side effects were also determined. Moreover, the sampled population was also asked regarding people who are vulnerable to LF, what usually happens to a person with LF, and whether LF could be prevented or not. Corresponding scores were similarly assigned to responses on the knowledge questions. Knowledge summary scores were determined and categorized as low, moderate and high. Tests for association between potential factors and receipt of antifilarial drugs, as well as MDA acceptance were performed using Pearson chi-squared test corrected for survey design [9]. This study used a design-based inference (i.e. the associate inferences are based on the probability distribution induced by the sampling design with the population values held fixed) [11] from the standpoint of survey sampling, and did not utilize the classical model-based inference where the sampling design (i.e. how the sample was selected) plays little role and inference stems entirely from a superpopulation model (a model which includes a random component is responsible for creating the elements in the population) [12]."

The level of significance used in the univariate tests of association was 10%. A higher value than the usual 5% level of significance was considered so that the initial analysis would be more lenient in retaining variables that may be subsequently included in the multivariate analysis.

Responses from the qualitative surveys, key informant interviews and focused group discussions, were transcribed, coded, and encoded. Themes were extracted from the responses and results were triangulated with quantitative results in order to enhance confidence in the ensuing findings [13], to contextualize the survey results and formulate recommendations.

## Results

### Socio-demographic Characteristics

The survey included 437 respondents. The mean age of the sampled population was 40 years old (standard error 1.2 years), while the median did not vary markedly at 39 years old with a range of 18 to 78 years old (Table 1). A smaller number of male than female respondents were interviewed and post-stratification sampling weights were applied to achieve a male to female ratio of 1.08: 1. More than three fourths of the sampled population was married, and almost all had attended school. Among those who attended school, nearly half completed or had studied but were not able to finish elementary. Three fourths of the sampled population had sources of income, with farming as the most common occupation. Abaca or banana farming was the source of income of 8.5% of the sampled population.

**Table 1:** Socio-demographic characteristics of the sampled population, Agusan del Sur, Philippines, 2005

**Socio-demographic characteristics**		**Percent**
MEAN AGE (year), SE MEDIAN AGE		40, 1.2
MEDIAN AGE		39
SEX	Male	51.9
	Female	48.1
CIVIL STATUS	Single, never married	15.1
	Currently married	77.4
	Currently living w/someone	0.2
	Divorced/separated	0.8
	Widowed	6.4
EDUCATIONAL ATTAINMENT	Elementary level/graduate	49.4
	High school level/graduate	31.3
	College level/graduate	14.4
	Vocational level/graduate	3.1
	No education	1.9

### Receipt of Antifilarial drugs

At the time of the survey, the study areas had already completed the second round of MDA. Some municipalities started the MDA in July 2004, while others started in October of the same year.

Almost two-thirds (63.3%) of the sampled population reported that they were able to receive antifilarial drugs. Of these, 78% received the drugs at a designated fixed point within the *barangay *[e.g. *barangay *hall, *barangay *health station, waiting shed, or multi-purpose hall] or municipality [e.g. rural health unit, public market, gymnasium, school]. More than one-fifth (21.6%) of the sampled population said that the volunteer health workers distributed the drugs to their houses or workplaces during the mopping up activity. Others were excluded from taking the drugs because of certain health conditions such as hypertension and pregnancy.

### Intake of Antifilarial drugs

Among those who received the drugs, nearly all (94.5%) reported having ingested them. When asked why they took the drugs, a majority reasoned that intake of the drugs would prevent LF. Some (8.9%) ingested the drugs because they believed that they might be infected with LF and wanted to get well.

The health workers' concept that MDA (which they popularly call "mass treatment") prevents and reduces transmission of LF is consistent with the sampled population's belief regarding MDA. However, some health workers have reasoned that intake of antifilarial drugs could treat hydrocoele.

Twenty-six percent of the sampled population took the drugs because they believed in the health workers' advice, which again is supported by the qualitative results describing the role of health workers in the dissemination of information about mass treatment.

The majority (91.2%) of the sampled population participating in the MDA reported that they took the drugs on a full stomach as advised by the health workers. Only a small minority (2.1%) ingested the drugs on an empty stomach. When asked who witnessed their drug intake, 44.4% said that they were seen by their household members, 38.3% by the *barangay *health workers, 27.6% by their friends, neighbours or co-workers, and 8% by the rural health unit staff.

Among those who participated in the MDA, 5.2% did not ingest all the drugs that are part of the combination therapy. Some ingested either diethylcarbamazine alone or albendazole alone, while others refused to take any of the drugs they received. The most common reasons for not ingesting the two drugs were fear of side effects and having forgotten to take them. These replies were further verified during the focused group discussions where drug intake refusal was mainly due to the reported adverse drug reactions such as drowsiness, headache, abdominal pain, and vomiting. Also, misconceptions about adverse effects caused by the drugs (e.g., sterility; fainting, and even death), and inability to work efficiently were reasons for refusal. Some male respondents refused to take the drugs because they would have to abstain from alcohol drinking and cigarette smoking.

When asked to specify who would decide whether to take the drugs or not, 64.7% of the sampled population said that they would decide on their own. Others cited certain people who would influence their decision, such as health workers (12.5%), parents (3.8%), and spouses (1.8%).

Health workers from all sampled municipalities reported in the focus group discussions that the MDA in 2004 had higher treatment coverage than the previous year, except in one municipality. In this particular municipality, the municipal health officer was physically present during the MDA implementation in all *barangays *in 2003 but not so in the following round. The decrease in coverage was also attributed to the reported adverse drug reactions.

Community leaders and health workers were motivated to reach the target coverage due to the reported decrease in the number of positive cases in their community, the increase in people's awareness and knowledge of the disease, and the clarification of various misconceptions regarding antifilarial drugs, which resulted in an increase in MDA treatment coverage.

### Awareness about LF and MDA

Ninety two percent of the sampled population claimed to have heard the term "filariasis". They expressed that they first heard it from a television program and video presentation, and from neighbouring *barangays *that had positive cases. They associated the term with enlarged legs, feet, scrotum, and breasts. Some health workers believed that it was a mosquito-borne disease and others expressed that the disease is contagious. In one municipality, the health workers reported that some community members believed that rheumatism, witchcraft, and exposure to hot and cold could cause LF.

The majority (89.1%) of the sampled population claimed to have heard of "mass treatment" or "MDA". Of these, almost all (92.1%) believed that the purpose of MDA was for LF. Of the sampled population, 17.8% mentioned that mass treatment was conducted for schistosomiasis, 5.2% and 2.8% said that it was for malaria and deworming, respectively. In addition, 7.1% mentioned that MDA was for other purposes such as vitamin supplementation, immunization, tuberculosis, dengue, swelling, goiter, and other diseases. A small proportion of the sampled population (2.6%) had no idea about the purpose of the MDA.

### Knowledge on LF

Both the key informant interviews and the survey results showed that mass media, specifically television and video, was the community's primary source of information about LF (49% of the sampled population). Other forms of mass media such as pamphlets, flyers and posters (29.9%) and radio (15.8%) were also identified as sources of information. Meanwhile, common interpersonal sources of information on LF included *barangay *health workers (46.0%), rural health unit staff (36.3%), neighbours (19.0%), and co-workers and people from other towns (9.5%).

Sixty-three percent of the sampled population believed that LF was caused by mosquito bites. Only 1.1% mentioned that LF was caused by parasites, microbes or intestinal worms, while 0.9% knew that LF could be acquired whilst working or staying in the abaca or banana plantations. More than one-fourth of the sampled population (28.7%) did not know the cause of the disease.

The most common manifestation of LF mentioned by the sampled population was the enlargement of body parts such as scrotum and female genitalia (58.7%), legs and feet (54.1%), breasts (52.9%), and arms (28.8%) (Table 2). These figures may explain why the sampled population associated the word filariasis with enlarged body parts. Only a small minority (0.2%) said that infected patients could be asymptomatic. One-fifth of the sampled population did not know any symptoms of LF.

**Table 2:** Knowledge on the clinical manifestations of lymphatic filariasis of the sampled population, Agusan del Sur, Philippines, 2005

Clinical manifestations of lymphatic filariasis*	**Percent**
Enlarged scrotum/vulva	58.7
Elephantiasis/enlarged legs and feet	54.1
Enlarged breast	52.9
Enlarged arms and hands	28.8
Fever	11.5
Weakness	8.2
Headache	4.8
Abdominal pain	3.2
Enlarged part where mosquito bit	2.8
Chills	2.6
Dizziness/vertigo	0.8
Reduced appetite	0.5
Nausea/vomiting	0.3
Enlarged lymphnodes	0.2
Can be asymptomatic	0.2
Others	7.6
Don't know	19.5

*with multiple responses

More than half (56.0%) of the sampled population said that LF could be diagnosed through physical examination, which was mostly performed by a physician and occasionally by a rural health unit staff or any health worker. Only 10.4% knew that LF could be diagnosed through blood examination while 29.8% did not know how LF could be diagnosed.

When asked about the treatment for LF, 64.1% of the sampled population knew that LF could be treated with tablets, but they could not specify the name of the tablets. Both diethylcarbamazine and albendazole were mentioned as treatment for LF by only 0.9% of the sampled population. Thirty-three percent did not know what the treatment for LF was.

The majority (82.4%) of the sampled population believed that LF could be prevented. They mentioned MDA as the most common form of prevention (48.0%), while others believed that taking medications (11.0%) or antifilarials (3.6%) could prevent the disease. Some cited that LF could be prevented by using mosquito control methods in general (29.0%), and by specific methods such as use of mosquito nets (17.6%), cleaning of the surroundings (13.2%), and spraying of insecticides (2.1%).

Thirteen percent of the sampled population believed that LF could not be prevented. Of these, 25.3% argued that this was so because mosquitoes are impossible to eliminate. Moreover, 11.2% said that they were not sure who would be bitten by the parasite-carrying mosquito and who would later get infected. There were also those (9.0%) who said that the disease is naturally endemic in the area posing everybody at risk of infection, hence it could not be prevented.

One-third (33.9%) of the sampled population said that the treatment for LF had no side effects, while 29% believed otherwise. Of the latter, almost half (46.2%) learned about the side effects from their own experience, 34.5% learned from their neighbours and 4.4% learned from doctors, teachers, classmates and friends. Dizziness or nausea was the most common side effect mentioned by the sampled population, followed by headache, and body weakness (Table 3). Meanwhile, 37% did not know whether or not the treatment has side effects.

**Table 3:** Knowledge on the side effects of treatment for lymphatic filariasis of the sampled population, Agusan del Sur, Philippines, 2005

Side effects of treatment for lymphatic filariasis*	**Percent**
Dizziness/nausea	18.0
Headache	7.4
Body weakness	5.6
Vomiting	4.7
Abdominal pain	4.3
Sleepiness	3.5
Rashes/itchiness	2.7
Fever	1.7
Difficulty in breathing/chest pain	0.5
Diarrhea	0.3
Chills	0.2
Others	1.0

*with multiple responses

The information, education and communication materials found at the health centres were mostly about LF and were not specific for MDA for LF. Also, these materials were written either in English or Tagalog language. In this regard, the health workers expressed the need for information, education and communication materials, which focus on the MDA strategy for LF, and the need for these materials to be translated into the local dialect.

### Attitudes

Fear was the first thing that came to mind in 35.4% of the sampled population when they heard the word "filariasis." Some associated it with disease (25.1%), while some related it with elephantiasis (14.1%) or enlargement of the scrotum (24.3%), breasts (18.9%), and arms and hands (6.5%). Others (7.2%) answered that they felt nervous when they heard the word filariasis, while some said that filariasis was deadly. Health workers perceived the problem on LF as dangerous and could evoke fear knowing that there were reported cases in their municipalities. They attributed their fear to their belief that LF is a serious disease, to the uncertainty of being bitten by filaria-carrying mosquitoes, and to the consequences of being infected with LF. They believed that having the disease would result in inability to work and earn a living, physical deformity, social stigma, and not being able to find a partner or get married. The sampled population also expressed the abovementioned attitudes towards LF (Table 4).

**Table 4:** Perception of the sampled population towards lymphatic filariasis (LF) and mass drug administration, Agusan del Sur, Philippines, 2005

**Attitude statements**	**Agree (%)**	**Disagree (%)**
**Perceived susceptibility**		
"LF is a part of life here in our town/*barangay*"	49.3	48.6
"Protecting myself and my family from LF is my responsibility"	97.4	0.5
**Perceived severity**		
"I am not worried about LF because it is not fatal"	22.3	75.4
"LF is a serious disease"	91.7	6.0
"It is hard for people with LF to work/earn a living"	92.9	4.7
"People with LF cannot find partners (husbands/wives)"	72.4	25.0
"People with LF are ashamed of their condition"	88.3	9.1
"I pity people with LF"	96.3	0.3
**Perceived benefits minus perceived barriers**		
"It would be easier for me to just go to the nearest health facility for diagnosis and treatment when I feel ill"	96.7	1.2
"The protection provided by antifilarial drugs are not worth the side effects"	16.8	80.8
"The protection provided by antifilarial drugs are not worth the inconvenience of taking them"	3.9	93.9
**Perceived threat**		
"I might be infected with LF now"	31.5	66.4
"I might be infected with LF in the future"	50.5	46.6
"Members of my family might be infected with LF now"	31.8	66.1
"Members of my family might be infected with LF in the future"	49.0	48.8
**Perception of government services**		
" The *Barangay *Health Station staff provides good services"	97.1	0.7
"The *Barangay *Health Station staff are always available for consultation"	96.9	1.0
"The rural health unit provides good service"	96.4	1.2
"The rural health unit staff are always available for consultation"	97.2	0.7
**Cues to action**		
"I should not have to take antifilarial drugs if I have no symptoms of LF"	28.5	69.2
**Theory of reasoned action**		
"I believe I am physically capable of ingesting all the antifilarial drugs"	94.6	3.2
"Ingesting all the antifilarial drugs will prevent me from getting LF"	97.2	0.7

The majority of the sampled population agreed on the following: it is their responsibility to protect themselves and their families from the disease, the risk of side effects was worth taking given the protection afforded by MDA and the inconvenience of taking the pills. Although half of the sampled population were equivocal of the possibility of infection in the future, the majority disagreed of the possibility of them or their families being infected at present.

Almost all of the sampled population expressed that it was not difficult for them to go to the nearest health facility for diagnosis and treatment when they feel ill. The majority agreed that the *barangay *health station and rural health unit provide good service and their staff are always available for consultation.

Nearly all the sampled population believed that they were physically capable of ingesting all the antifilarial drugs and that these drugs would prevent them from being infected. However, 28.5% of the sampled population mentioned that they should not take the antifilarial drugs if they are asymptomatic.

The majority were willing to take the antifilarial drugs in the next MDA round in order to prevent the disease (71.1%) or to be treated in case of infection (20.3%).

### Factors associated with receiving the antifilarial drugs

The sex of the respondents was found to be significantly associated with receiving the drugs with more females receiving the drugs than their male counterparts (Table 5). Age group, civil status, and level of education were not significantly associated with drug receipt.

**Table 5:** Association of factors with the receipt of antifilarial drugs, Agusan del Sur, Philippines, 2005

***Factors***		**Receipt of antifilarial drugs**		**p-value**
			
		**Yes (%)**	**No (%)**	
**Socio-demographic characteristics**				

Sex	Male	58.0	42.0	0.01
	Female	69.0	31.0	
Civil Status	Never married, single	95.9	4.1	0.60
	Ever married	93.9	6.1	
Education	Elementary level or lower	100.0	0.0	0.65
	High school or higher	94.6	5.4	

**Awareness**				

Awareness on lymphatic filariasis	Yes	66.2	33.8	0.01*
	No	28.4	71.6	
Awareness on MDA	Yes	66.5	33.5	0.02*
	No	36.9	63.1	
Awareness on MDA for lymphatic filariasis	Yes	69.1	30.9	0.01*
	No	36.4	63.6	

**Knowledge**				

Knowledge on lymphatic filariasis	Moderate	70.5	29.5	0.08*
	Low	59.6	40.4	

**Accessibility**				

Accessibility (in terms of distance)	RHU^a ^and BHS^b^	66.5	33.5	0.22
	RHU^a ^only	100.0	0.0	
	BHS^b ^only	50.9	49.1	
	Not accessible	67.0	33.0	
Accessibility (in terms of clinic hours)	RHU^a ^and BHS^b^	64.2	35.8	0.20
	RHU^a ^only	100.0	0.0	
	BHS^b ^only	37.3	62.7	
	Not accessible	75.0	25.0	

*Significant association, ^a^RHU-rural health unit, ^b^BHS-barangay health station

Awareness about LF, MDA, and MDA for LF were found to be associated with the receipt of antifilarial drugs. A higher proportion of those who were aware about LF, MDA, and MDA for LF received the drugs compared to those who were not aware. Knowledge on LF was also found to be associated with drug receipt. A higher proportion of those who had moderate level of knowledge received the drugs compared to those with low level of knowledge. Access to health facilities, in terms of distance and clinic hours, were not associated with receipt of antifilarial drugs.

### Factors associated with MDA acceptance

Socio-demographic characteristics (sex, age, civil status, religion, education and household income) and awareness of LF, MDA and MDA for LF were not significantly associated with MDA acceptance (Table 6). However, knowledge on LF was significantly associated with MDA acceptance. A higher proportion of those who received the drugs with moderate level of knowledge ingested the drugs compared with those who had low level of knowledge. Other factors such as being able to discuss LF and MDA with other people and being encouraged by others to take the drugs were not associated with MDA acceptance.

**Table 6:** Association of factors with mass drug administration (MDA) acceptance, Agusan del Sur, Philippines, 2005

***Factors***		**MDA acceptance (Intake of all MDA drugs)**		**p-value**
			
		**Yes (%)**	**No (%)**	
**Socio-demographic characteristics**				

Sex	Male	95.9	4.1	0.60
	Female	93.9	6.1	
Civil status	Single	95.0	5.0	0.59
	Ever married	93.3	6.7	
Education	Elementary level or lower	93.6	6.4	0.48
	High school or higher	95.9	4.1	

**Awareness**				

Awareness on lymphatic filariasis	Yes	94.6	5.4	0.52
	No	100	0.0	
Awareness on MDA	Yes	94.5	5.5	0.46
	No	100	0.0	
Awareness on MDA for lymphatic Filariasis	Yes	94.2	5.8	0.46
	No	100.0	0.0	

**Knowledge**				

Knowledge on lymphatic filariasis	Moderate	97.1	2.9	0.08*
	Low	93.5	6.5	

**Attitude**				

Perceived susceptibility to lymphatic Filariasis	High	94.5	5.5	0.38
	Low	100.0	0.0	
Perceived severity of lymphatic Filariasis	High	94.8	5.2	0.61
	Low	100.0	0.0	
Perceived benefits of antifilarial drugs	High	96.8	3.2	0.06*
	Low	90.6	9.4	
Perceived threat of lymphatic filariasis infection	High	97.2	2.8	0.16
	Low	93.3	6.7	
Attitude towards MDA	High	94.8	5.2	0.81
	Low	100.0	0.0	

*Significant association

The reliability coefficient alpha obtained for the attitude scale was 76%. The perceived benefits of antifilarial drugs (the protection provided by the drugs was considered worth the side effects and inconvenience of taking them) were significantly associated with drug intake (Table 6).

People's perceived susceptibility to LF, severity of LF, threat of LF, perception that an individual who is asymptomatic should not take the drugs, and attitude towards MDA were not significantly associated with acceptance.

The sampled population had a good perception of government services (i.e. good services at the rural health unit and *barangay *health station, and availability of health staff for consultation). Accessibility of the rural health unit and *barangay *health station, in terms of distance and clinic hours, was not associated with MDA acceptance.

## Discussion

The MDA therapeutic coverage in Agusan del Sur in 2004 was reported to be high, at 92% [14]. The respondents in this survey consisted of individuals aged 18 years old and above, hence the estimated coverage cannot be directly compared to the reported coverage. However, even if 100% of the younger population below 18 years (constituting approximately 52% of the total population of Agusan del Sur [15]) had been treated, the maximum achievable coverage in the province would have been 81%, indicative of a possible discrepancy (of at least 11%) between the reported and the actual MDA coverage. However, if the coverage rate in the younger population were the same as that of the sampled population, then the coverage rate would have been 60%. This estimated coverage is 32% lower than the reported coverage and it appears that the target coverage of 85% of the eligible population set by the Department of Health may still have not been reached.

In a World Health Organization report, the LYMPHASIM model [16] showed that the probability of LF elimination depends on a nonlinear fashion on treatment coverage, number of treatment rounds and endemicity level. Based on the model, if an area has a microfilaremia prevalence of 10%, and 80% of the population is covered at each round, four to five rounds will be sufficient to achieve elimination. However, if treatment coverage is 60%, then at least nine rounds will be needed to achieve elimination. Swaminathan *et. al *(2008) also described other factors that may influence LF elimination such as efficacy of the drugs of choice, their mode of action, and the possibility of developing drug resistance; the role of vector-parasite combinations; the magnitude of transmission thresholds; host-parasite interactions and their effects on the dynamics of infection and immunity; parasite biology, and progression to LF-associated disease [17]. The estimated surveyed coverage rate of Agusan del Sur is low, and if based solely on the LYMPHASIM model, the LF elimination in the province may take longer than the expected five years or five rounds of MDA. Furthermore, the duration of LF elimination in the province may either be as projected by the LYMPHASIM model assuming that the abovementioned factors present in the province favour LF elimination or it may even take much longer when the conditions do not bolster an environment conducive for LF elimination.

In the province, it has been reported that the antifilarial drugs were mainly distributed through fixed sites. However, results of the study showed that only half of the sampled population (49.4%, that is, 78% who received the drugs from the fixed site out of the 63.3% sampled population who received the drugs) submitted to MDA through this distribution strategy. The other half of the sampled population needed to be mopped up. However, only 13% (that is, 21.6% who received the drugs through house-to-house distribution out of the 63.3% of the sampled population who received the drugs) was reached. This result is not similar to the findings of Weerasooriya *et al*. where the majority of their sampled population received the drugs through house-to-house delivery [18]. In a report on optimization of interventions, it was shown that centralized drug distribution through community health centres and schools achieved maximum coverage [19]. However, in this study, the low proportion of the sampled population who went to the fixed sites may be due to the insufficient knowledge and awareness on MDA for LF. If the whole community were well informed, there may be an increase in submission to MDA through fixed sites. Moreover, delivery of drugs may be ensured if infrastructure of fixed sites such as that of the school system and local government units would be optimized, through a more systematic approach and empowerment of their constituents.

The World Health Organization-developed Communication for Behavioural Impact strategy, which advocated for house-to-house drug distribution, was also reported to increase MDA coverage in other endemic areas [2]. Although this strategy is the recommended approach, its application to the study site may need to be reassessed. In this study, mopping up activity contributed a minimal increase in the coverage and was difficult to implement, especially in far-flung areas. This was also compounded by the fact that the population density (62 per square kilometre) of the province was very low and forestland constitutes 76% of its total land area. [20].

The majority of the sampled population who personally received the drugs allotted for them ingested the drugs. Health workers witnessed the drug intake of less than half of the sampled population. However, there were cases where one household member received the drugs allotted for their households either through house-to-house visits by the health workers or through fixed sites. In both ways, the ingestion of the antifilarial drugs may not have been witnessed by the health workers or the drugs may not have been ingested at all. Hence, the discrepancy between the reported and the surveyed coverage may be attributed to the assumption that the number of people given the drugs is equal to the number of people who actually took the drugs. The drugs may not have been ingested due to various reasons such as household members were sick at the time, or were not screened for eligibility and decided not to take the drugs; refused to take the drugs; forgot to take the drugs, and were not well-informed about MDA. Direct observation of drug intake may help ensure that the reported coverage represents a reliable estimate of the proportion of the eligibles that actually ingest the drugs, instead of the proportion of the eligibles that receive the drugs.

Among the other basic information on LF, transmission of the disease through mosquitoes was most widely known by the sampled population. Although the association of people's knowledge on LF transmission with MDA acceptance was not directly assessed, a study by Mathieu *et al*. found that knowledge on transmission of LF by mosquitoes was positively associated with taking the antifilarial drugs [21]. This may help explain the results that nearly all who received the drugs reported to have ingested them.

In disease entities such as depression, substance abuse, physical disabilities and stressful life events, men are less likely to seek help from health professionals than women [22]. In the case of filariasis, for which most signs and symptoms do not appear at the earlier stages of the disease, it can be expected that women would fair better than men in submitting to mass treatment as shown in this study.

Based on the Health Belief Model [5], when perceived benefits outweigh the perceived barriers, people are more likely to comply with MDA. Moreover, increased knowledge about the drugs and their side effects may result in a better perception of its benefits than its barriers. Consistent with the Health Belief Model, the study results showed that the protection provided by the drugs was perceived to be beneficial and significantly associated with drug intake.

Nearly all those sampled did not know that a person with LF could be asymptomatic. The majority were only aware of the manifestations of the disease, which appear in its later stages. This lack of knowledge may have influenced their health-seeking behaviour such as going to the fixed site to receive the drugs and their perception of being infected, especially when they do not have symptoms and do not feel ill.

Only a small proportion of the sampled population knew that the diagnosis of LF was through blood examination. This result may be due to the limited number of *barangays *where surveillance through nocturnal blood examination was conducted. The majority of the community members knew that LF could be diagnosed through physical examination and not through blood examination. Given this, people need to be informed about the life cycle of filarial worms, that an infected person can be asymptomatic, that anyone could be infected, and that the symptoms of the disease may appear five to ten years after being infected [23]. This may motivate the community members to submit in the MDA even though they are asymptomatic and have not been diagnosed through nocturnal blood examination.

Almost all those interviewed did not know the name of the antifilarial drugs given during the MDA. Health workers should keep in mind that community members have the right to information and it is their ethical responsibility to explain such details. Although the study has shown that people in the community may not be very particular with details, such as the name of the drugs, it is more important that the people would receive and ingest the drugs to help achieve the desired outcome of increased MDA coverage.

The majority of the sampled population expressed their willingness to participate and ingest the drugs in the next round of MDA. This may be attributed to their perceived threat of becoming infected with LF in the future, as well as their knowledge on LF prevention. The people's attitude towards behaviour (intake of drugs) may be seen in in their claim of being physically capable of ingesting all the antifilarial drugs. This shows that the challenge posed by ingesting numerous antifilarials drugs during MDA may be considered manageable.

In this study, a low incidence of side effects was reported, and no severe adverse events were experienced. These findings were similar to the results of Molyneux and Zagaria (2002), where treatment with antifilarial drugs did not lead to any severe adverse events and that the combination of diethylcarbamazine and albendazole was safe and offered little cause for concern. They mentioned that the side effects seen with antifilarial drugs were all mild, and seem to be related with its therapeutic effects [24].

The lack of knowledge on side effects of a large proportion of the sampled population may later affect the success of the MDA as it has been reported that side effects of antifilarial treatment is a major factor in decreased compliance in the second and subsequent years of MDA for LF elimination [25]. Submission to MDA results in decreased prevalence and intensity of LF infection. Adverse events result from drug effects on existing infections and may indicate that the drugs are taking effect. The greater the intensity of infection (meaning more worms), the greater may be the side effects. There is a need therefore to more effectively promote MDA through information, education and communication so that targeted people in spite of adverse events will submit to MDA.

In this study, some community members who were not eligible (e.g pregnant women or lactating mothers) were found to have received the antifilarial drugs. This indicates that the eligibility criteria for mass treatment may not have been strictly followed. In addition to preparing implementers of MDA in the field, health workers may have to be given refresher courses that emphasize screening for treatment eligibility, effectiveness and safety of antifilarial drugs, counselling and adverse drug reaction management.

The study showed that mass media, specifically television, was widely utilized and shown to be crucial in disseminating information about LF and MDA. Moreover, health workers remain to be the community's major source of information on LF, indicating that their active and sustained participation is vital in running a five-year MDA programme to eliminate LF. The efforts of health workers may also need to be complemented with continuing if not intensified support from the local government unit.

In 2004, the Office of the President of the Republic of the Philippines released the Executive Order 369 [26], stating that Filariasis Month will be observed every November, during which MDA for LF would be implemented in endemic areas. However, during the survey, community leaders lacked awareness about Executive Order 369, and there were no local ordinances or policies drafted for the implementation of the MDA in Agusan del Sur. This reflects that MDA may not have been backed up with sufficient groundwork, and the desired buy-in of implementers at the local government may not have been achieved [27]. Even with lack of awareness, the community leaders still showed support for the MDA by participating in activities such as social preparation, information dissemination, and drug distribution. However, increased awareness of the Executive Order 369 could have elicited more support from the local government units in terms of planning and budget allocation.

Health education and promotion activities, utilizing locally translated information, education and communication materials and other media, may increase the community's level of awareness and knowledge on LF and MDA and heighten their understanding of the benefits of antifilarial drugs and participation in MDA, resulting in increased treatment coverage and eventual elimination of LF.

## Conclusion

In conclusion, a low proportion of the sampled population in Agusan del Sur received and ingested the antifilarial drugs. A discrepancy between the reported and the surveyed MDA coverage was observed. Only half of the sampled population received the drugs through a fixed site, while the labour-intensive house-to-house strategy yielded a minimal additional increase in treatment coverage. These strategies should be reassessed in terms of feasibility and cost in order to achieve the desired coverage. People's knowledge and awareness on MDA for LF as well as their perceived benefits of the antifilarial drugs should be improved through an intensified health education and promotion activities. Sufficient groundwork for MDA and buy-in from implementation partners, specifically from the local government should be achieved to produce tangible results. These findings may be utilized to improve MDA implementation, which would help increase treatment coverage, and ultimately, contribute to the successful elimination of LF in the province.

## Competing interests

The authors declare that they have no competing interests.

## Authors' contributions

MLA participated in the design of the study, acquisition of data, statistical analysis, and data interpretation and writing of the paper.

VYB participated in the design of the study, data interpretation and writing of the paper.

JTS participated in the data interpretation and writing of the paper.

SMS participated in the design of the study, acquisition of data, and data interpretation and writing of the paper.

ASD was involved in the acquisition of data and writing of the paper.

All authors were involved in drafting the manuscript and have given approval of the version to be published.
